# Integration of photomagnetic bimodal imaging to monitor an autogenous exosome loaded platform: unveiling strong targeted retention effects for guiding the photothermal and magnetothermal therapy in a mouse prostate cancer model

**DOI:** 10.1186/s12951-024-02704-0

**Published:** 2024-07-17

**Authors:** Songlu Liu, Wenting Shang, Jian Song, Qiubai Li, Liang Wang

**Affiliations:** 1 Department of Radiology, Beijing Friendship Hospital, Capital Medical University, Beijing, 100050 China; 2 CAS Key Laboratory of Molecular Imaging, Beijing Key Laboratory of Molecular Imaging, Beijing, 100190 China; 3 Department of Urology, Beijing Friendship Hospital, Capital Medical University, Beijing, China; 4 Department of Radiology, University Hospitals Cleveland Medical Center, Cleveland, OH USA

**Keywords:** Prostate cancer, Magnetic particle imaging, Fluorescence molecular imaging, Exosome, Photothermal therapy, Magnetothermal therapy

## Abstract

**Background:**

Prostate cancer (PCa) is the most prevalent cancer among males, emphasizing the critical need for precise diagnosis and treatment to enhance patient prognosis. Recent studies have extensively utilized urine exosomes from patients with cancer for targeted delivery. This study aimed to employ highly sensitive magnetic particle imaging (MPI) and fluorescence molecular imaging (FMI) to monitor the targeted delivery of an exosome-loaded platform at the tumour site, offering insights into a potential combined photothermal and magnetic thermal therapy regime for PCa.

**Results:**

MPI and FMI were utilized to monitor the in vivo retention performance of exosomes in a prostate tumour mouse model. The exosome-loaded platform exhibited robust homologous targeting ability during imaging (SPIONs@EXO-Dye:66·48%±3·85%; Dye-SPIONs: 34·57%±7·55%, **P<0·01), as verified by in vitro imaging and in vitro tissue Prussian blue staining.

**Conclusions:**

The experimental data underscore the feasibility of using MPI for in vivo PCa imaging. Furthermore, the exosome-loaded platform may contribute to the precise diagnosis and treatment of PCa.

**Graphical Abstract:**

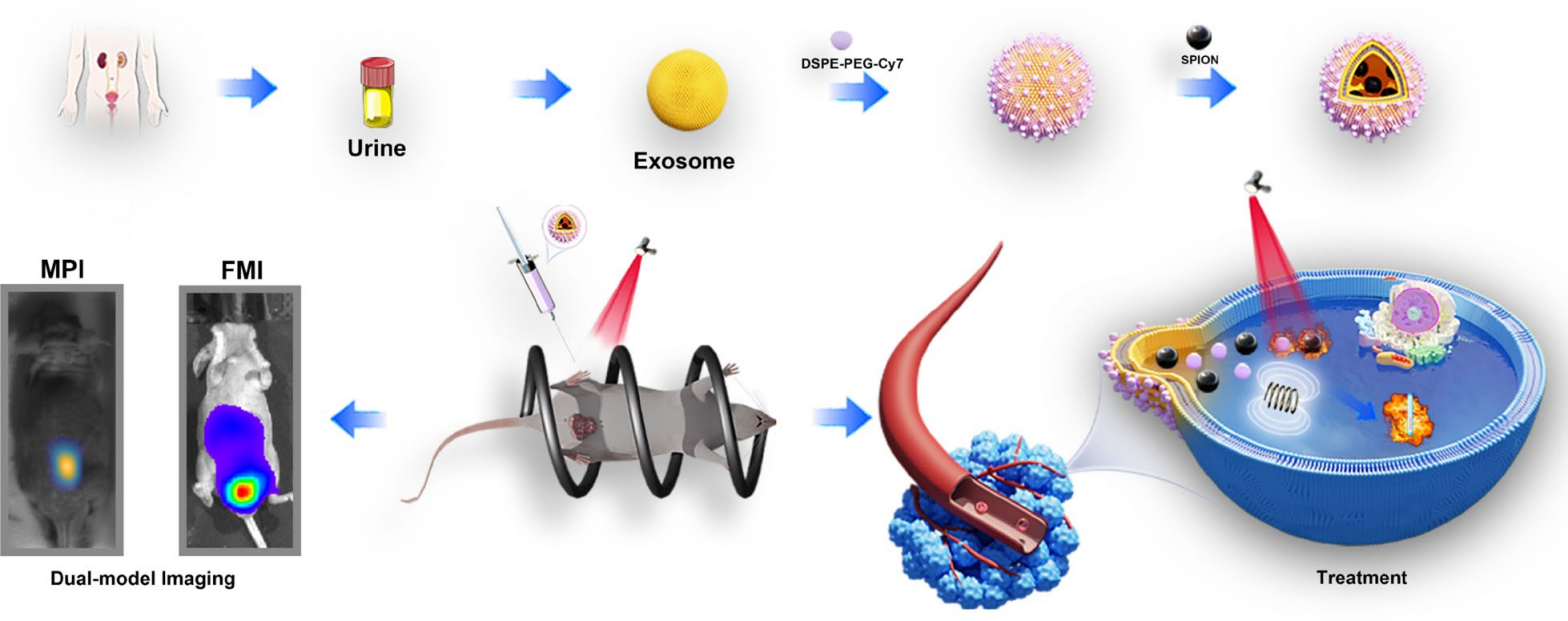

**Supplementary Information:**

The online version contains supplementary material available at 10.1186/s12951-024-02704-0.

## Introduction

Prostate cancer (PCa) poses a significant health challenge affecting millions of men globally, comprising 27% of new cancer diagnoses in men worldwide, with an estimated 268,490 new cases in men in 2022 [1]. Its slow early-stage progression can escalate to metastatic castration-resistant PCa or neuroendocrine PCa, resulting in a grim prognosis [2]. With an 11% mortality rate in 2022, PCa stands as the second leading cause of cancer death in men, necessitating precise diagnosis and therapy for improved prognoses and survival rates.

Targeted imaging and precision therapy for PCa require specific PCa targets [3]. Prostate-specific membrane antigen (PSMA) stands out as a prevalent molecular imaging target for PCa diagnosis, given its heightened expression in PCa [4–6]. The effectiveness of ^68^Ga-PSMA PET/CT has been validated for detecting primary prostate cancer [7, 8] Nonetheless, the presence of PSMA receptors in certain normal tissues poses a challenge, potentially leading to false-positive results during imaging [6, 9, 10]. This highlights the need for an enhanced targeting platform. Exosomes, nanoscale single-membrane vesicles (30 to 200 nm) derived from cells, emerge as promising entities with distinctive attributes that position them as excellent drug carriers [11–14]. Their remarkable features include high biocompatibility, efficient transcellular permeability, natural circulatory stability, and low immunogenicity and immunotoxicity. These characteristics collectively contribute to their suitability for drug delivery applications. The robust cellular uptake and targeted homing capabilities of exosomes open avenues for synthesizing biomimetic nanoparticles tailored for tumour targeting. This versatility elevates exosomes above conventional nanomaterials, rendering them suitable for targeted metabolic imaging [14–16]. Traditionally, the isolation of exosomes from the tumour cell culture medium yields an exceptionally low quantity, rendering them unsuitable for clinical application [15–17]. Recent advancements have pivoted towards extracting exosomes directly from urine samples of tumour patients, presenting distinct advantages such as higher yields and obviating the need for extensive cell culture [16–18]. Urine-derived exosomes, enriched with surface proteins like CD9 and CD47, serve as carriers for the diagnosis and treatment of PCa. Notably, CD9 plays a pivotal role in facilitating the fusion of exosome membrane structures with target cells, enhancing the cellular delivery of therapeutic agents, whereas CD47 acts as a shield against phagocytosis by the immune system [17, 18].This strategic use of exosomes from urine samples not only improves practicality but also enhances their efficacy in PCa applications.

While Magnetic Resonance Imaging (MRI) remains a prevalent tool for precise detection, biopsy guidance, and local staging of PCa, its limitations, particularly its low specificity leading to false-positives in low-risk patients, prompt the exploration of alternative imaging modalities [19, 20]. Positron Emission Tomography with Prostate-Specific Membrane Antigen (PSMA-PET), although promising for PCa staging, suffers from challenges such as low specificity and false-positives, and radiation risks [5, 6, 9, 10]. In response to these challenges, there arises a critical need for highly sensitive and high spatially precise imaging technologies. Magnetic particle imaging (MPI) emerges as a novel, non-invasive method for real-time quantitative imaging, utilizing superparamagnetic iron oxide nanoparticles (SPIONs) distributed throughout living organisms [21, 22]. Notably, the exclusive detection of signals in SPIONs results in an exceptionally high contrast-to-noise ratio without background interference [23]. Complementing MPI, fluorescence molecular imaging (FMI) brings high sensitivity and spatial resolution, making it a valuable tool in the diagnosis and treatment of PCa [24, 25]. However, FMI’s limitation in imaging depth calls for innovation solution. Integrating MPI with FMI, leveraging their combined strengths, offers a synergistic approach for achieving high-resolution and highly sensitive imaging but also establish a reliable method for detecting exosome-loading platforms in vivo. The amalgamation of these advanced imaging modalities holds the promise of enhancing precision in PCa diagnostics and therapeutic monitoring.

Superparamagnetic iron oxide nanoparticles (SPIONs) have garnered significant attention in the realm of MPI, emerging as valuable agents for tumor imaging in various studies [22, 26, 27]. Beyond their imaging prowess, SPIONs have robust magnetic heating properties, rendering them suitable for tumour magnetic heating treatments. Notably, magnetic hyperthermia, a distinctive feature of SPIONs, transcends the limitatons of depth encountered in photothermal therapy, exhibiting the capability to heat deep-seated tumours [28]. A key player in multimodal approach is Cyanine 7 (Cy7), a versatile component widely used in molecular imaging and therapeutics. Cy7’s appeal lies in its negligible fluorescent background and an enduring imaging period within the spectral range of 700–1000 nm [29–31]. Furthermore, Cy7’s unique to absorb near-infrared light and convert it into heat positions as a valuable asset for photothermal therapy [32, 33].

**Scheme 1:**
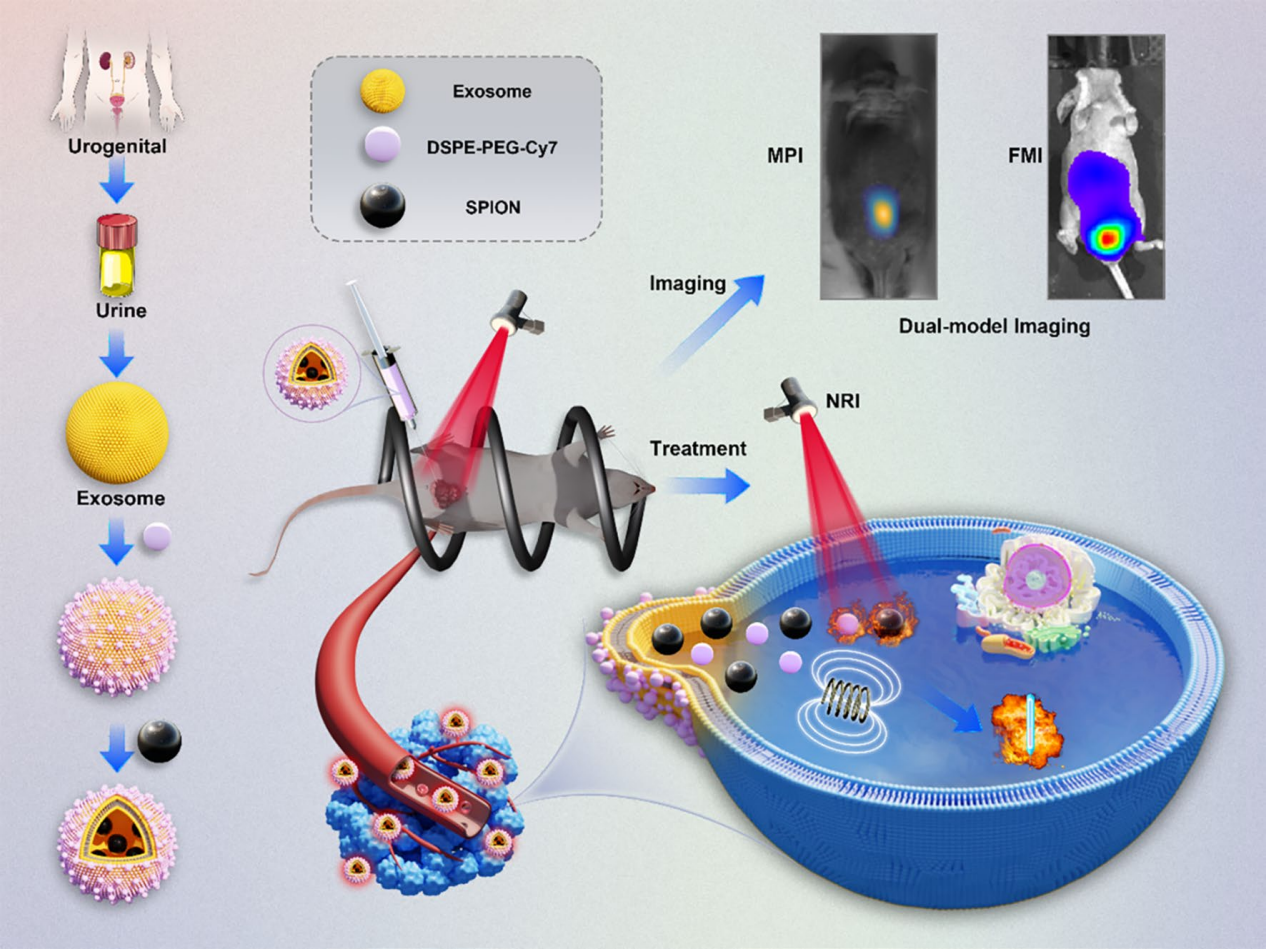
Schematic of metabolic imaging of the constructed exosome-loaded platform, denoted as SED, sourced from prostate cancer (PCa) patients. The metabolic imaging is conducted in orthotopic and subcutaneous PCa models, employing dual-modality imaging techniques. Additionally, the schematic outlines the application of thermotherapy for PCa leveraging the unique characteristics of the developed platform

In this study, we harnessed the unique attributes of urine-derived exosomes from patients with PCa, recognizing their exceptional versatility as carriers. Leveraging the inherent qualities of exosomes, we designated them as a nanoparticle loading platform, introducing polyethylene glycol (PEG) stable SPIONs and fluorescent dye Cy7 into the exosome nanoparticles, and named SPIONs@EXO-dye (SED). Notably, these probes synthesised in this way exhibit both FMI and MPI signals, presenting an innovative dual-modal imaging capability. This dual functionality was leveraged to monitor exosome metabolism at tumour sites, providing compelling evidence of the targeted retention effect of exosomes. Moving beyond imaging, our study ventured into therapeutic realms, employing a synergistic approach of photothermal and magnetothermal therapy. Through precise thermal treatments applied both superficially and deep within the tumour, we demonstrated the efficacy of our exosome-supported platforms. This study not only validates the targeted retention effect of exosome but also underscores the potential of utilising exosome’s targeted binding properties in the realm of combined photothermal and magnetothermal therapy for PCa. The integration of advanced imaging and therapeutic strategies presented herein contributes significantly to the evolving landscape of precision medicine for PCa (see Scheme 1).

## Results

### Characterisation of the exosome-loading platform

**Fig. 1:**
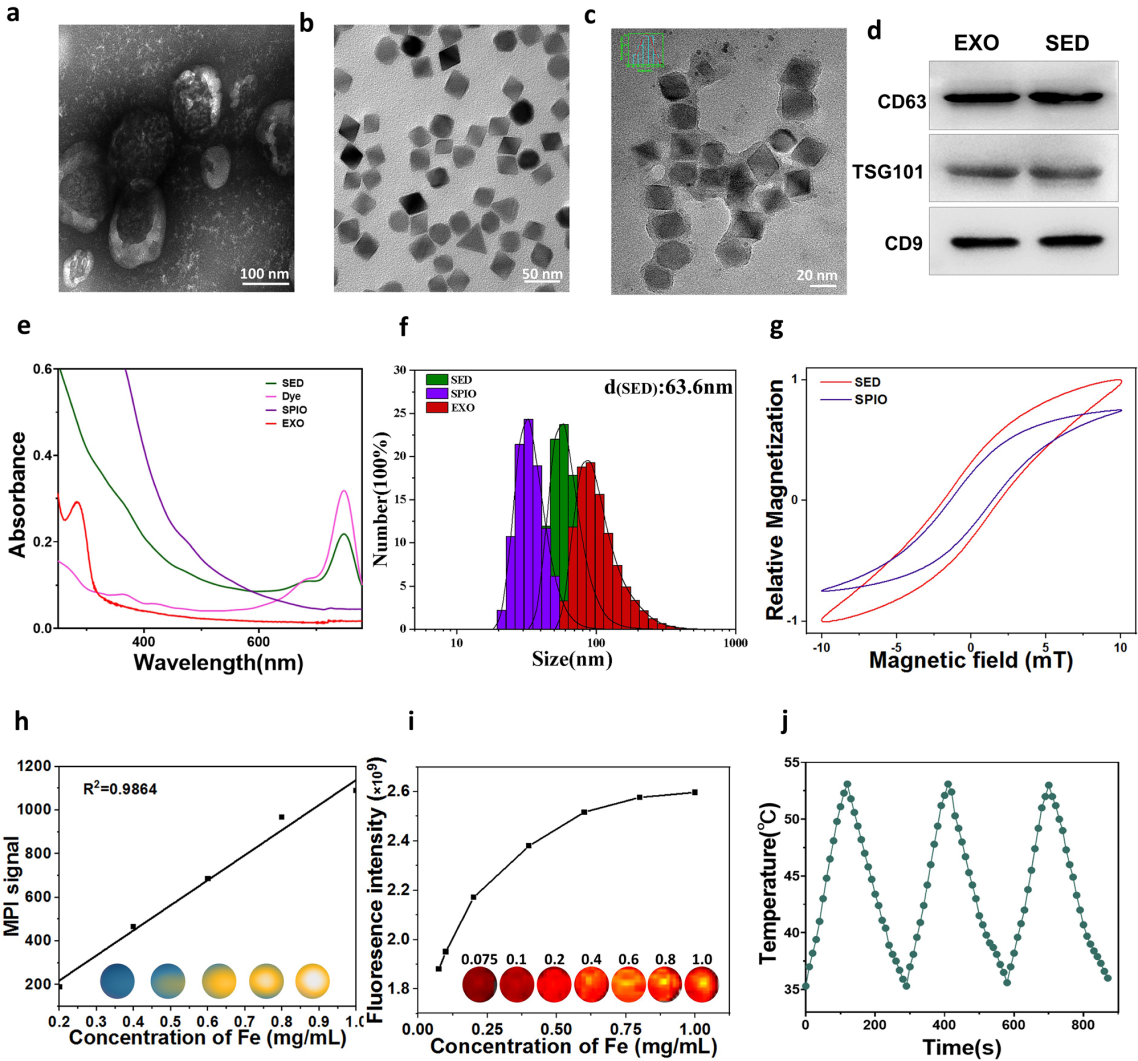
Characterisation of the SED exosome loading platform. Transmission electron microscopy images of (**a**) EXO, (**b**) SPIO, and (**c**) SED nanoparticles. Scale bar a: 100 nm; b: 50 nm; c:20 nm. (**d**) Western blot analysis of CD9, CD63 and TSG101 in EXO and SED. (**e**) Absorbance spectrum of SED nanoparticles. (**f**) Analysis of hydrated particle size in SED exosome-loaded platform. (**g**) Hysteresis loop of SED and SPIO. (**h**) Magnetic particle imaging (MPI) signal of SED with varing iron concentration. (**i**) IVIS spectra depicting that the fluorescence intensity of SED increased with varing fluorophore concentration. (**j**) Photothermal swing curve of SED nanoparticles

**Fig. 2:**
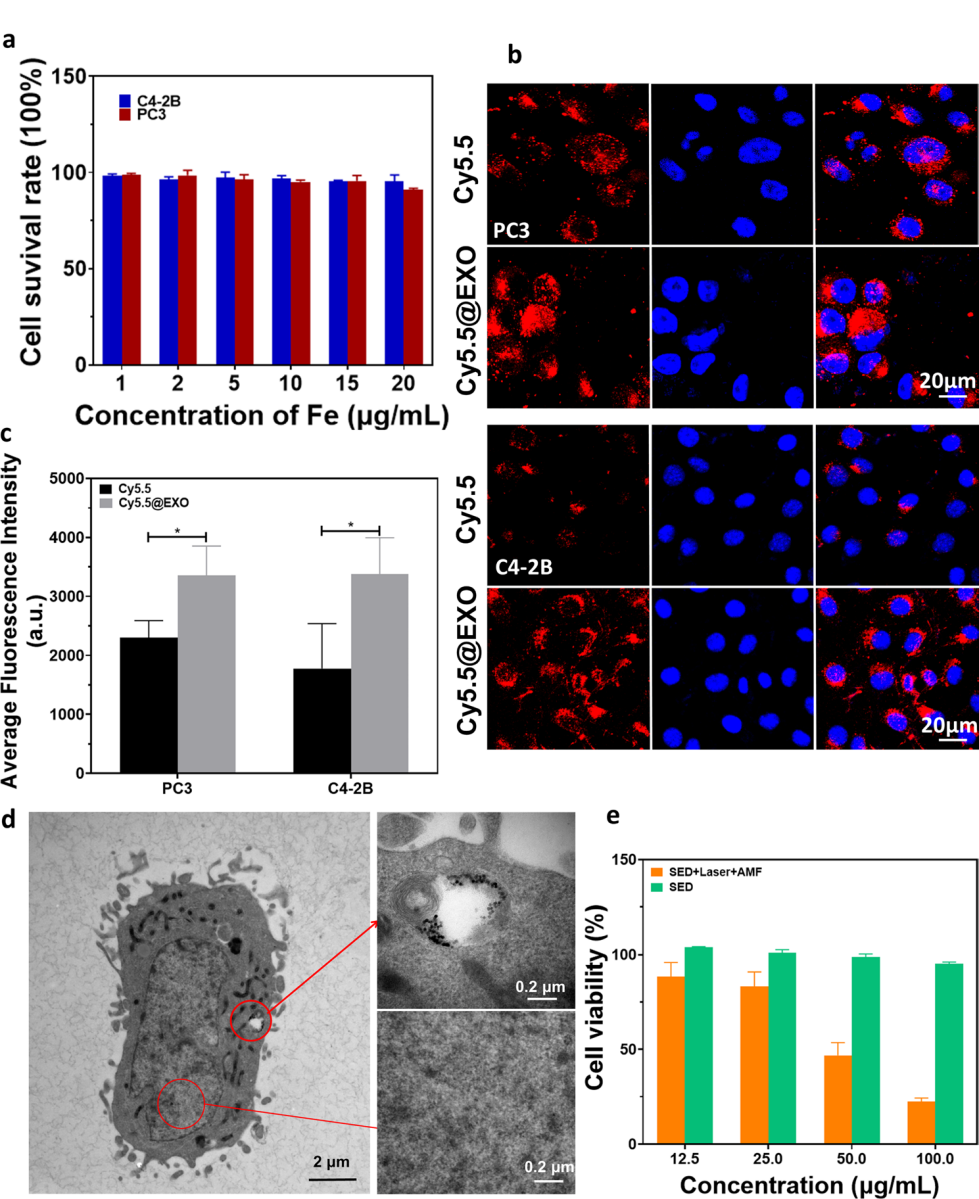
Cytotoxicity test of the exosome-loaded platform and the exosome targeting test. (**a**) Effects on the viability of PC3 and C4-2B cells with increasing concentrations of the exosome-loaded platform SED. (**b**) Cy5.5 and Cy5.5@EXO were incubated with PC3 and C4-2B cells, with images taken by laser scanning confocal microscopy (scale bar: 20 μm). (**c**) Quantitative analysis of images taken by confocal microscopy. (**d**) Biological electron microscopy images of PC3 cells taking up exosome loaded platform (scale bar: 2 μm, 0.2 μm). (**e**) Effect of SED photothermal and magnetic thermal treatments on the viability of PC3 cells

In this study, exosomes were extracted from urine samples obtained from patients with PCa. The loading of fluorescent dye and SPIONs into the exosomes was accomplished through a combination of sonication and co-incubation, followed by the removal of excess impurities via centrifugation. Transmission electron microscopy (TEM) images were captured to illustrate the structure of SPIONs and exosomes (Fig. 1a, b).TEM observations further showed that the SED nanoparticles maintained a uniform size of 33 nm (Fig. 1c), with the average hydrated particle size of 63.6 nm (Fig. 1f). The nanoparticles embedding within the exosomes was confirmed through TEM images of the nanoparticles and exosome-loaded platforms (Fig. 1a, b, c). Western blot analysis affirmed that the exosome-loaded platform and exosomes exhibited identical protein profiles for CD63, CD9, and TSG101 expressions, indicating that the exosome-loaded nanoparticles did not affect these characteristic proteins (Fig. 1d). The UV–visible broad spectrum characteristics of the samples are depicted in Fig. 2e. Urine exosomes exhibited a faint absorption peak around 290 nm, corresponding to the liposome absorption peak. In contrast, both Cy7 and SED consistently exhibited their highest absorption peaks at approximately 750 nm. Simultaneously, the magnetisation characteristics of the magnetic particles were assessed using magnetic particle spectroscopy (MPS), revealing the sustained strong magnetisation characteristics of the exosome-loaded platform (Fig. 1g). Zeta potential testing across various materials unveiled that the final zeta potential of the exosome-loaded platform was approximately − 32·6 mV [see Additional file: Figure S1a]. Notably, the hydrated particle size of SED in DMEM medium, containing 10% serum, remained stable over seven days, indicating robust colloidal stability of SED. [see Additional file: Figure S5b].

### In vitro FMI/MPI performance of exosome-loading platforms

To evaluate the FMI and MPI performances of the exosome-loaded platform, we conducted an initial validation of its in vitro imaging performance. SED solutions with different concentrations were prepared using different iron concentrations. At lower iron concentrations, the fluorescence signal exhibited a positive correlation with the sample concentration. The MPI signal, on the other hand, demonstrated a positive and linear correlation with the sample iron concentration, as illustrated in Fig. 1h. However, with increasing sample concentration, the fluorescence exhibited a decrease, leading to a non-linear correlation between fluorescence intensity and sample concentration (Fig. 1i). Meanwhile, SED exhibited commendable thermal stability, as indicated in Fig. 1j. These findings collectively underscore the versatile in vitro imaging performance and thermal stability of the exosome-loaded platform.

### In vitro targeting and cytotoxicity of exosome-loaded platforms

In the initial phase, the cells were cultured in a mixed solution of the probe and medium at different concentrations for 24 h, and subsequent cell activity was tested using the CCK-8 colorimetric method (Fig. 2a). The results depicted in the figure indicate that the probe had no significant toxicity to the two cell lines under investigation. For further validation of targeting specificity, Cy5.5@EXO and Cy5.5 were co-incubated with the PCa cell lines PC-3 and C4-2B, respectively. Confocal images revealed a markedly higher uptake of Cy5.5@EXO by the two PCa cell lines compared to Cy5.5 alone, affirming the targeting specificity of the exosome-loading platform in PCa cells (Fig. 2b, c). Human umbilical vein endothelial cells (HUVEC) were employed as the control group, demonstrating minimal uptake Cy5.5@EXO under the same experimental conditions. This further confirmed the targeting capability of autogenous exosomes towards prostate cancer cells [Additional file: Figure S1d]. To observe the uptake of SPIONs by the cell lines post-incubation with the probe, biological electron microscopy captured the cellular uptake primarily within the cytoplasm (Fig. 2d). Additionally, the therapeutic effect of SED was validated at the cellular level by co-incubation, followed by laser irradiation and exposure to a magnetic field (Fig. 2e). These findings collectively demonstrate the targeted uptake and therapeutic potential of the exosome-loaded platform at the cellular level.

### In vivo MPI and FMI dual-modality imaging in a subcutaneously injected mouse model

**Fig. 3:**
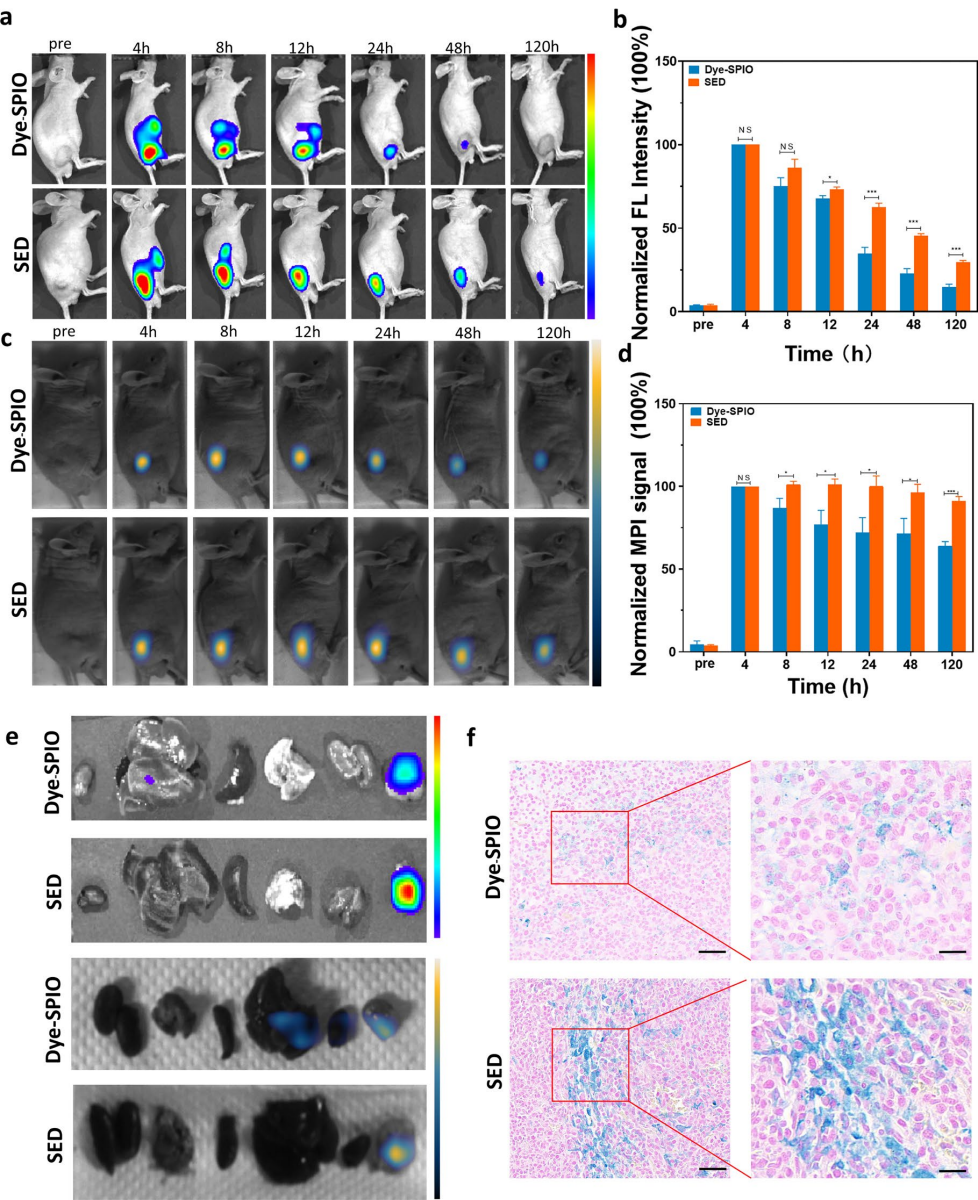
Dual-modality imaging of a subcutaneous PC3 model. (**A**) Fluorescence images of the PC3 subcutaneous model at different time points. (*n* = 4). (**B**) Quantitative comparison of normalized fluorescence intensities. (**C**) MPI images of the subcutaneously injected PC3 mouse model at different time points (*n* = 4). (**D**) Quantitative comparison of normalized MPI signals. (**E**) In vitro fluorescence imaging and in vitro MPI images of tumour tissues and major organ tissues of two groups of PC3 subcutaneous tumour models. (**F**) Comparison of Prussian blue staining of tumour tissues between the two groups of PC3 subcutaneous tumour models. Student t test: **, *P* < 0.001; **, *P* < 0.01; Scale bar in the miniaturized image: 50 μm, scale bar in the enlarged image: 20 μm

To assess the specific targeting effect of the exosome-loaded platform on PCa cells in vivo, we initiated FMI in a PC-3 subcutaneous tumour model (*n* = 4). By employing tail vein injection of Cy5.5 @EXOs (experiment group, EG) and Cy5.5 (control group, CG), the mice were imaged at various time points [see Additional file: Figure S2a] and the tumour signal background ratio (tumour normal ratio, TNR) was analysed. The obtained data revealed a peak TNR in the EG group at 24 h, approximately 1·74 ± 0·04, while the TNR of group CG at this time was 1·2 ± 0·05 (***P < 0·001) [see Additional file: Figure S2b, c]. Subsequent imaging at different times demonstrated clear distinction of tumours in the EG group, whereas the mice in the CG did not visibly outline the tumour. Finally, the mice were anatomised [see Additional file: Figure S2d]. Fluorescence signal comparison between tumours and crucial organs and tissues demonstrated significantly higher tumour signals in the EG compared to the CG (**P < 0·01) [see Additional file: Figure S2e], along with notably lower liver signals in the EG, affirming the targeting of the exosome-loaded platform. To enhance therapeutic efficacy, we replaced the fluorescent dye with Cy7, monitoring the targeted retention characteristics of exosomes at the tumour site using MPI and loaded SPIONs for magnetic heat therapy. In the experimental group, PC-3 subcutaneous tumour mice were injected with SED intratumorally, while the control group received Dye-SPIONs. The FMI results revealed that a peak fluorescence intensity 4 h post-injection,, with both groups exhibiting mostly identical fluorescence intensities at this time (Fig. 3a). The fluorescence intensity of the experimental and control groups decreased gradually 4 h post-injection, with the experimental group consistently displaying higher fluorescence intensity at different time points. Statistical analysis confirmed these results (Fig. 3b), with a significant difference in standardized fluorescence intensity observed at 12 h (experimental group: 73·18%±1·44%, control group: 67·64%±1·87%, *P < 0·05), and the most noticeable difference at 120 h (experimental group: 29·7%±2·01%; control group: 14·71%±1·78%). This demonstrates the potent retention property of the exosome-loading platform at the tumour site, closely related to the targeting ability of exosomes and interaction between exosomes and tumour cells.

The more sensitive MPI test substantiated this conclusion, with imaging of the experimental and control groups at 4, 8, 12, 24, 48, and 120 h after injection (Fig. 3c), consistent with the FMI results. Standardised calculation of the MPI signal revealed a difference 8 h after injection (Fig. 3d) (experimental group: 101·10%±1·93%, control group: 86·78%±5·92%, *P < 0·05), and the disparity persisted (experimental group: 91·26%±2·55%, control group: 63·97%±2·60%, ***P < 0·001). The robust retention properties of the exosome-loaded platform were further demonstrated.

To validate the efficacy of the probe, we conducted ex vivo imaging of the mouse tumour and major tissues and organs 5 days post-injection, assessing signal intensity. FMI of the control and experimental groups (Fig. 5e) revealed a significant difference in tumour signals, with the experimental group exhibiting higher signals under identical conditions. Subsequent histological analysis and Prussian blue staining were performed on the tumour tissues and major organs (Fig. 3e). As shown in Fig. 3f, Prussian blue staining of the tumour tissues in the SED group was more intense than that in the Dye-SPIONs group, and the positive part was more widely distributed, confirming the presence of more SPIONs in tumour tissues in the experimental group. This result underscores the targeted homing effect of the exosome-loaded platform. Additionally, we also observed that the Prussian blue-positive rates in the liver and spleen tissues in the SED group were lower than those in the control group [see Additional file: Figure S3], further confirming the efficacy our results.

### In vivo dual-modality imaging of FMI and MPI in the orthotopic mouse model

**Fig. 4:**
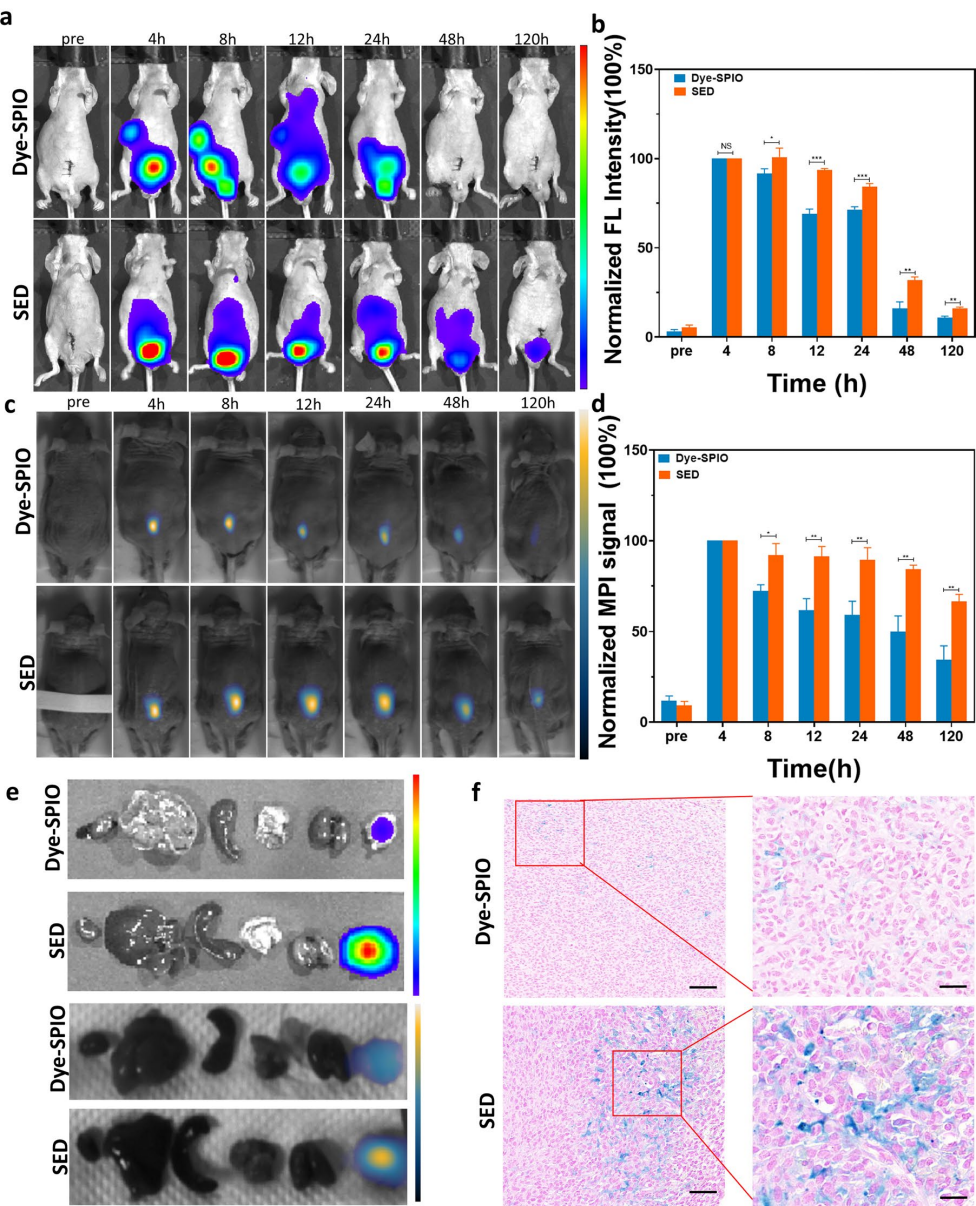
Dual-modality imaging of the PC3 orthotopic model. (**A**) Fluorescence images of the PC3 in situ model at different time points. (*n* = 4). (**B**) Quantitative comparison of normalized fluorescence intensities. (**C**) MPI images of orthotopic PC3 mouse models at different time points (*n* = 4). (**D**) Quantitative comparison of normalized MPI signals. (**E**) In vitro fluorescence imaging and in vitro MPI images of tumour tissues and major organ tissues of two groups of orthotopic PC3 tumour models. (**F**) Comparison of Prussian blue staining of tumour tissues between the two groups of orthotopic PC3 tumour models. Student *t* test. **, *P* < 0.001; **, *P* < 0.01; Scale bar in the miniaturized image: 50 μm, scale bar in the enlarged image: 20 μm

The orthotopic tumour model serves as an effective simulator of the tumour microenvironment. To validate this, we performed imaging using an orthotopic PCa mouse model, established with PC-3 cells [see Additional file Figure S1b] and utilized FMI and MPI for imaging in this orthotopic PCa model. Notably, the fluorescence signals of other tissues in FMI were significantly disrupted, possibly due to the limited imaging depth and light scattering issues inherent to FMI. To overcome these challenges, MPI was used to monitor the metabolic distribution of the exosome-loaded platform at the tumor site. Initially, orthotopic mouse models were randomly assigned to two groups, receiving injections of SED or Dye-SPIONs nanoparticles into the tumour (*n* = 4). The fluorescence intensity in both the experimental and control groups peaked at 4 h post-injection (Fig. 4a), consistent with the subcutaneous tumour model. After 4 h, the fluorescence intensities gradually decreased, with the SED group exhibiting a slower decrease than the control group, indicating enhanced enrichment of the exosome-loaded platform in the tumour site of the treatment group. The discrepancy reached its peak at 12 h after injection (Fig. 4b) (experimental group: 93·5%±0·96%, control group: 69·10%±2·64%, ***P < 0·001). Monitoring the fluorescence signal intensity over 120 h after injection, the discrepancy in the strength of the fluorescence signal persisted, with the signal of the tumour site in the SED group remaining stronger than that in the Dye-SPIONs group (Fig. 4b) (experimental group: 31·90%±1·81%, control group: 15·82%±3·04%, **P < 0·01). Notably, the fluorescence signals of other tissues in FMI were significantly disrupted, possibly due to the limited imaging depth and light scattering issues inherent to FMI. To overcome these challenges, MPI was used to monitor the metabolic distribution of the exosome-loading platform at the tumour site.

In vivo MPI results (Fig. 4c) demonstrated that MPI signal of the tumour site in the control and experimental groups peaked at approximately 4 h after injection; however, the MPI signal at the tumour site in the control group clearly decreased faster than that in the experimental group. This proved that the enrichment effect of the exosome-loading platform at the tumour site was considerably stronger than that in the control group, and this conclusion was confirmed following the standardised calculation of MPI signals at different time points (Fig. 4d). As shown in the figure, the MPI signals in the experimental group differed from those in the control group at 4 h after injection (experimental group: 91·85%±6·54%, control group: 72·15%±3·52%, *P < 0·05), and differences were still observed at 120 h after injection (experimental group: 66·48%±3·85%, control group: 34·57%±7·55%, **P<0·01).

To verify these conclusions, we collected tumour tissues and major organs from the experimental and control groups 120 h after injection and performed ex vivo imaging using FMI and MPI to detect the signal intensity of different tissues (Fig. 4e). In vitro FMI analysis results confirmed a higher fluorescence signal in the experimental group, supporting the targeted retention effect of exosomes. ex vivo MPI results were consistent with those of FMI, confirming the targeting effect of the exosome-loaded platform on tumour cells and its retention effect at the tumour site. Prussian blue staining of the tumour tissues in the experimental group was notably stronger and more widely distributed (Fig. 4f), consistent with metabolically important tissues [see Additional file: Figure S4].

### Photothermal/magnetic thermal properties of exosome-loaded platform & anti-tumour properties in vivo

**Fig. 5:**
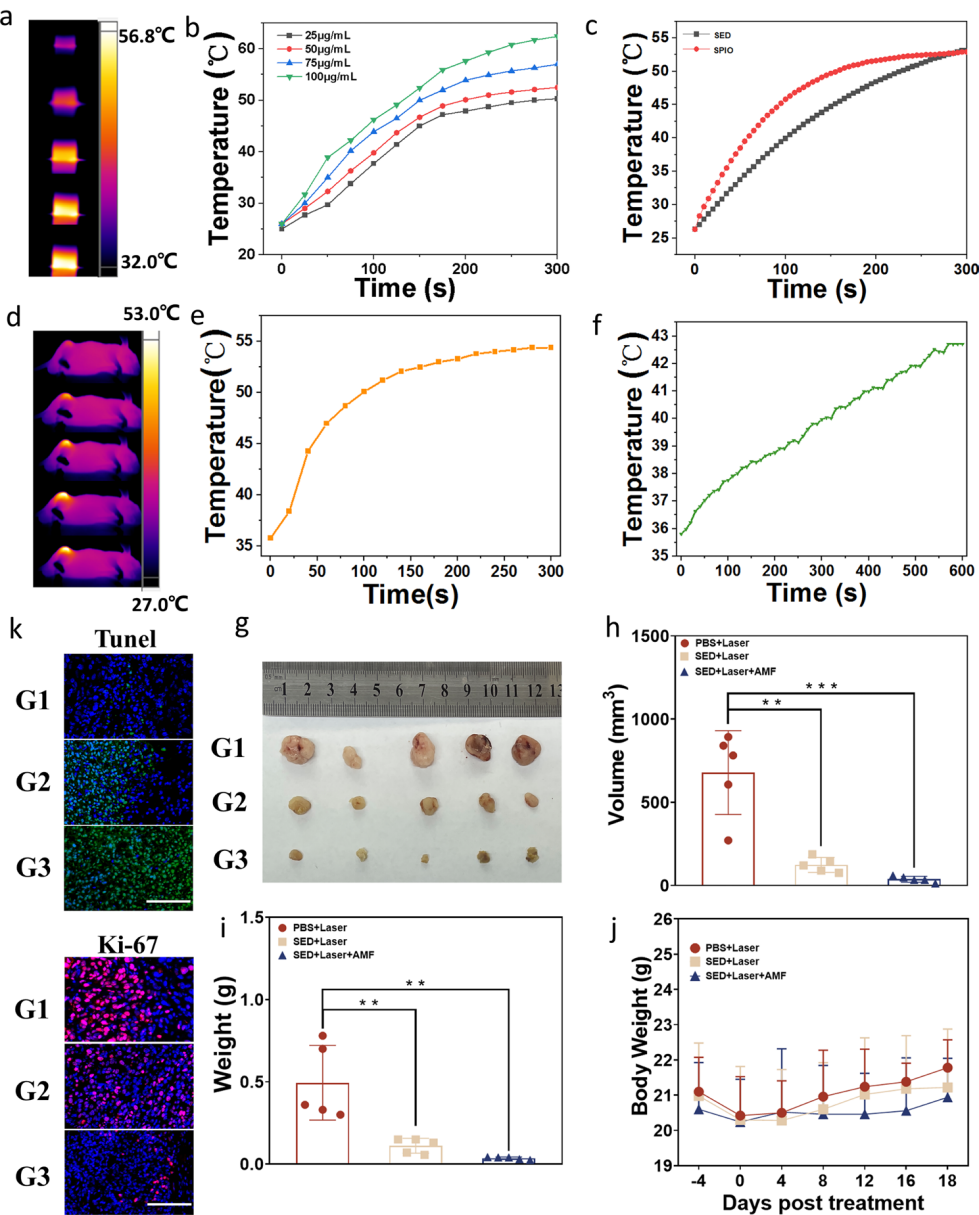
Thermotherapeutic effect of exosome-loaded platform SED. (**a**) Thermal infrared image of SED under laser irradiation. (**b**) Temperature changes of different concentrations of SED under laser irradiation for 5 min. (**c**) Temperature changes of SED and SPIO in the presence of a magnetic field. (**d**) Infrared thermography of SED in vivo photothermal therapy. (**e**)SED in vivo photothermal therapy temperature change curve. (**f**) SED in vivo magnetic heating treatment temperature change curve. (**g**) Ex vivo photographs of tumours under different treatment groups. (**h**)(**i**) Tumour volume and tumour weight for different treatment groups. (**j**) Curves of body weight change of mice in different treatment groups. (**k**) TUNEL and Ki-67 fluorescence in tumour sections of representative mice from different groups. Scale bar: 100 μm

The in vitro photothermal and magnetothermal properties of the exosome-loaded platforms were initially examined. Photothermal treatment involved the use of near infrared (NIR) light (785 nm, 1.2 W/cm^2^) was used for 5 min (Fig. 5a), with simultaneous monitoring of temperature changes in materials with different concentrations. Photothermal images of materials with varying concentrations are shown in Fig. 5b. For magnetic heating, an alternating magnetic field of 354 kHz and 30 A was applied to the material, and the temperature changes in the SPIONs and exosome-loading platform were measured using a temperature-measuring fibre (Fig. 5c). The exosome-loading platform exhibited comparable magnetic heating effect to SPIONs during the magnetic field support.

The experimental mice in G1, G2, and G3 groups were then exposed to laser light, with the experimental protocol outlined in a flow chart[see Additional file: Figure S5a].The thermal imaging map of mice during photothermal therapy is shown in Fig. 5d, and the temperature change curve of photothermal therapy in Fig. 5e. The tumour site reached a temperature of 54·2 °C after 5 min of laser irradiation. Subsequently, in vivo magnetic heating was performed on G3 mice, with temperature changes monitored using temperature-measuring optical fibres (Fig. 5f). The temperature of mice reached 42·7 °C under the influence of a magnetic field. Tumor volumes in different groups of mice were monitored during treatment [see Additional file: Figure S5c]. After treatment, the tumour tissues of the mice were dissected and photographed (Fig. 5g). Comparative analysis of tumour volumes in the different groups revealed that the G3 group exhibited the lowest tumour volume, while the G1 group showed the highest. This indicated that SED + Laser + AMF yielded optimal therapeutic efficacy, likely due to the strong permeability and prolonged retention of the exosome-loading platform. Quantitative analyses of the tumour volume and weight are shown in Fig. 5h and i, respectively. The data obtained from the quantitative analysis were consistent with our observations, proving that the superior outcomes in the G3 group. In addition, mouse weights were measured during the treatment period, showing no significant difference in the weights of the mice, indicating high tolerance to the experimental process (Fig. 5j). The invasiveness and apoptosis rate of the three groups of tumour tissues were then evaluated. Tumour invasiveness was verified using Ki-67 immunofluorescence, and apoptosis was assessed using TUNEL immunofluorescence (Fig. 5k). Following the treatment, the G3 group demonstrated the optimal treatment effect, with the G1 group exhibiting minimum apoptosis and the G3 group having the lowest Ki-67 index, while the G1 group had the highest Ki-67 index. Histopathological changes were further evaluated using haematoxylin and eosin staining [see Additional file: Figure S5].

## Discussion

In this study, exosomes sourced from the urine samples of patients with PCa were used to synthesise an exosome-loaded SED platform. Using exosomes as carriers of nanoparticles, a dual-modality imaging approach utilizing MPI and FMI was employed to detect the accumulation and metabolism of the exosome-loaded platform at the tumour site.

MPI was specifically utilized for monitoring the metabolism of the exosome-loading platform in PCa and major organs. Notably, MPI, with its lack of depth limitations and minimal signal interference, compensating for the limitations of FMI, providing complementary and advantageous effects. The integration of these two imaging modalities enabled the acquisition of high-sensitivity and high-resolution imaging, enhancing the overall investigative capabilities of the study. FMI was employed to confirm the targeting proficiency of the exosome-loaded platform. Through tail vein injection imaging and subsequent quantitative analysis, a clear observation was made, highlighting the effective targeting of the subcutaneous tumor site by the exosome-loaded platform with homologous targeting.This platform demonstrated superior tumor imaging compared to the control group, exhibiting higher tumour signal-to-normal ratio. The study leveraged both FMI and MPI as dual-modality imaging techniques to monitor the metabolism and distribution of the exosome-loading platform at the tumour site and major organs.

The homologous targeting of the exosome-loaded platform, coupled with the interactions between tumour-derived exosomes and tumour cells, resulted in enhanced aggregation and retention rates of the SED at the tumour site, surpassing those of the control group. This outcome was confirmed through dual-modality imaging and quantitative analysis. Moreover, the exosome-loaded platform exhibited a more even distribution at the tumour site compared to the control, underscoring its high permeability and targeting precision. Concurrently, the photothermal and magnetic thermal properties of the SED were thoroughly examined. Photothermal and magnetic thermal treatments were administered to a subcutaneous tumour model, revealing that the exosome-loaded platform indeed possessed these treatment properties. Comparative analysis of tumor sizes among different groups of mice affirmed the efficacy of the exosome-loaded platform in photothermal and magnetic thermal treatments. To comprehensively evaluate the therapeutic effects, apoptosis and tumour invasion were assessed in the isolated tumour tissues. The results provided insights into the impact of the exosome-loaded platform on inducing apoptosis and inhibiting tumor invasion, further corroborating its potential for therapeutic applications.

In this study, the utilization of exosomes as carriers for synthesising nanomaterials proved to be a promising approach. Exosomes, derived from the intracellular compartments of eukaryotic cells, have gained considerable attention in clinical medical research due to their influence on cell communication, facilitated by their structure resembles to parental cell membrane [34]. The inherent homo-targeting property has sparked interest in using exosomes to synthesise targeted drug-delivery systems [35–37]. Previous studies have explored the use of exosomes to synthesise targeted imaging probes, including optical and photoacoustic imaging [37–39]. Notably, targeted exosomes carrying nanomaterials, and chemical reagents have been employed for the diagnose and treat gliomas, providing potential advancements in the diagnosis and treatment of intracranial tumours [39]. The study emphasizes the advantages of urine-derived exosomes, which exhibit higher productivity and lower immunogenicity compated to cell-derived exosomes. The investigation further validated the targeted retention property of the exosome-loaded platform via intratumoural injection of SED, which demonstrated exosome-cell interactions. Recently, intratumoural injections have gained prominence in diagnosing and treating PCa and other tumor types [40–42]. Experimental studies, such as the intratumoural injection of mimic miR-29b, have shown significant inhibition of PCa xenografts growth in nude mice [40]. Laprise-Pelletier et al. injected LDR AuNPs into PCa tumours and visualised them using multimodal imaging [41]. In a prospective randomised Phase II trial, an intratumoural tracer injection improved the identification of tumour-positive lymph nodes after prostatic (IP) tracer injection [42]. Intratumoural injections have also been explored for other tumour types, including a two-cohort pilot phase I study where patients with cancer received intratumoural image-guided 0·25 mg of the dsRNA analogue Hiltonol combined with radiation therapyIntratumoural injection of Hiltonol increased the expression of interferon-β and interferon-α in peripheral-blood mononuclear cells, showing stability and initial clinical efficacy of the combination therapy [43]. Systemic agents, when administered throughout the body, may lead to undesirable side effects and limit the use of optimal therapeutic doses.Intratumoural injections, on the other hand, involve directly delivering the therapeutic agents into the tumour, maintaining high concentrations at the tumour site and dissipating over time in the systemic circulation, present a metabolic approach bypasses the disadvantages of systemic administration and prevents systemic toxicity [44]. Intratumoural administration allows the drug access to tumour-draining lymph nodes [45]. The performed intratumoural injection of SED can be combined with multimodal imaging for image-guided imaging and treatment, benefiting from the homologous targeting of the exosome-loading platform and its long-term retention at the tumour site.

Iron oxide nanoparticles, known for their remarkable safety and clearance, have been extensively used in preclinical and clinical studies. They are suitable for multimodal MRI and MPI based on their characteristics [46]. The exosome-loading platform developed in this study utilized homo-targeting and low-immunogenicity exosomes as a carrier of nanomaterials. Loading them with a fluorescent dye and SPIONs generated dual-modality imaging, facilitating accurate tumours imaging.

Nano-iron oxide finds application in magnetic heating therapy [47]. Numerous studies have shown that magnetic heating can increase tumour permeability, creating a more sensitive environment for immunotherapy in solid tumours [48]. The constructed exosome-loaded platform, combining photothermal and magnetic heating, proves effective in treating tumours. Dual-mode photothermal and magnetic thermal therapies lead to tumour regression through the degeneration of collagen fibres and long-term thermal efficiency [49–52].

In summary, the exosome-loaded platform with dual-modality imaging features provides accurate localisation during image-guided therapy for combined photothermal magnetic hyperthermia at tumour sites. MPI, with its ability to observe the magnetic tracer without masking the signal of the background tissue, can be quantitatively combined with the local treatment of magnetic fluid hyperthermia (MFH), demonstrating high potential for developing precision treatments. Moreover, the selected exosomes, with homologous targeting, low immunogenicity, and biocompatibility in the human body, hold significant potential for clinical transformation.

## Methods

### Preparation of exosomes

Urine specimens were collected from fasting patients diagnosed with PCa in the morning and stored in a -80℃ refrigerator for subsequent exosome purification. Exosomes were extracted using an exosome purification kit (Beijing Omiget Pharmaceutical Technology Co., Ltd). Initially, a magnetic bead within a container was agitated for 30 s. A 2 mL urine sample was extracted, placed in a centrifuge tube, and centrifuged at 3000 g for 2 min at 4 °C. The supernatant was discarded, and magnetic beads were washed to eliminate any remaining supernatant. Subsequently, a mixture of Buffer EXP, Buffer EXN, Buffer EXT, and urine were configured, following the exosome purification system, and rotated at 4 °C for 1 h to collect the magnetic beads. The resulting mixture was combined with 4 mL of Buffer EXE, centrifuged at 7000 g at 4 °C for 2 min, and the upper suspension was transferred to an EP tube. After removing impurities through filtration, the supernatant was stored in a refrigerator at -80 °C.

### Preparation of exosome-loading platform

A 1 mL sample of exosome solution (1 mg/mL) were thoroughly mixed 1 mL of Cy5.5 solution (1 mg/mL) using an ultrasonic treatment. The integrity of the exosomes was deliberately disrupted by intermittently turning the ultrasonic instrument on and off every 30 s. This process was repeated thrice, and the obtained material was gently shaken for 2 h at 37 °C to restore the exosomes’ membrane. Cy5.5@EXO purified nanoparticles were obtained via centrifugation. Subsequently, DSPE-PEG Cy7 (Dye) was replaced with a fluorescent dye to enhabce photothermal efficacy. The same procedures were repeated to load DSPE-PEG-Cy7 and SPIONs into exosomes, of nanoparticle SPIONs@EXO-Dye (SED).

### Structural characterisation

The sizes and morphologies of the SPIONs and SED nanoparticles were examined using transmission electron microscopy (TEM, FEI Tecnai F20). UV–vis spectra of different materials were characterised using a UV–vis spectrophotometer. Hydrodynamic sizes and zeta potential were determined using Malvern Zetasizer (UK ZEN 3600). MPI signals were obtained using an MPI system (MOMENTUM, Magnetic Insight, USA). Solutions with iron concentrations ranging from 0·2, 0·4, 0·6, 0·8, and 1·0 mg/mL were prepared for MPI signals detection. FMI signal was acquired using an IVIS spectral imaging system (IVIS spectrum, PerkinElmer, USA). Iron concentrations of 0·075, 0·1, 0·2, 0·4, 0·6, 0·8, and 1·0 mg/mL were used to prepare solutions for FMI signal detection.

### Cell culture

Human PCa cell line PC3 cells stably transfected with the luciferase gene (PerkinElmer) were cultured in complete F12k medium in a cell incubator at 37 °C in 5% CO_2_ following the experimental protocol. The human PCa cell line, C4-2B, was cultured in basic RPMI 1640 medium supplemented with 10% FBS and 1% penicillin/streptomycin in the same culture environment as the PC-3 cell line.

### Animal experiments

BALB/c male nude mice (male, 6–8 weeks of age) were procured from Beijing Vitonglihua Experimental Animal Technology Co. Ltd. (China) and maintained under specific pathogens free conditions. The animals were carefully raised to ensure optimal health. To establish a subcutaneous tumour model for PCa, a 125 µL cell suspension was prepared using PCa cell lines PC-3 or C4-2B (2 × 10^6^), phosphate-buffered saline (PBS), and Matrigel. This cell mixture was then subcutaneously implanted in each nude mouse. To simulate the microenvironment of PCa, an orthotopic tumour model was established. Mice were anaesthetised, and a 2–3-mm incision was made in the midline above the pelvis. Using an insulin needle, a 50 µL cell suspension containing 1 × 10^6^ PCa cells was injected into the dorsal side of the prostate. The incision was carefully sutured with silk thread to facilitate proper healing. Upon reaching the predetermined subcutaneous tumour-bearing volume of approximately 100 mm^3^, the mice were divided into two groups. Subcutaneous and in vivo tumour-bearing mice were injected intravenously and caudally, and multimodal imaging procedures were conducted to facilitate comprehensive analysis and visualization of the tumour dynamics.

### Cytotoxicity of SED nanoparticles

PC3 and C4-2B cells were cultured (approximately 2000 cells/well) in 96-well plates and subjected to co-culturing with SED exosome-supported nanoparticles along with a control medium of varying concentration gradients. This incubation period lasted for 24 h. The cells were gently washed thrice with PBS to remove any residual nanoparticles or medium. For PC-3 cells, 100 µL of F12k medium containing 10 µL CCK-8 solution was added and allowed to incubate for 2 h. RPMI 1640 medium was used for C4-2B. Subsequently, the optical density was analysed at 450 nm, and the quantitative values were measured by enzyme labelling. This data was utilized to calculate the cell survival rate.

### In vitro targeting specificity assessment

A 100 µL cell(PC3,C4-2B, HUVEC) suspension (approximately 3 × 10^5^) and 1·5 mL culture medium were added to each well and incubated for 24 h. Fresh medium was replaced with Cy5.5 or Cy5.5@EXO at a concentration of 30 µg/mL, followed by incubation for 3–4 h. After gentle washing with PBS thrice, cells were fixed with 4% paraformaldehyde, stained with Hoechst under dark conditions, and observed via CLSM (LSM780, Carl Zeiss, Jena, Germany) and Thunder imager 3D assay imaging system(Leica Microsystems Ltd, Germany).

### Bioluminescence imaging

Orthotopic and subcutaneous changes in PCa were monitored via bioluminescence imaging (BLI) using an IVIS spectral imaging system (IVIS Spectrum, PerkinElmer, USA). A D-fluorescein solution was intraperitoneally injected, with a 6 to 8-minute interval, before in vivo imaging. BLI images were acquired by IVIS spectroscopy (PerkinElmer, Waltham, MA, USA) to provide a comprehensive view of the tumour site. Mice were anaesthetised during imaging process, ensuring minimal movement and optimal data acquisition. A 2% isoflurane/air gas mixture was administered to maintain the anesthesia.

### FMI

After injection of Cy5.5 or Cy5.5@EXO via the tail vein, mice were anaesthetised with isoflurane for FMI. Images were obtained using IVIS spectroscopy before and at 6, 8, 10, 12, 18, 24, and 48 h after injection. After euthanasia, major organs and tumour tissues were isolated for ex vivo imaging. Regions of interest (ROI) of the tumour and muscle areas were acquired to compute TNR, and signal intensity was quantified using IVIS Living Imaging 4·4 software. In addition, FMI was subsequently performed following an intra-tumour injection of Dye-SPIONs or SED in mice, and images were obtained before, and at intervals of 4, 8, 12, 24, 48, and 120 h after injection. All mice were analysed using standardised fluorescence intensity metrics. The mean fluorescence intensity ratio (TNR) of tumour to normal tissue at 4 h served as the baseline (100%), and the standard fluorescence intensity was determined as the ratio of TNR at different time points to TNR at 4 h. Ex vivo imaging of vital organs and tumour tissues were performed as previously described, contributing to a comprehensive understanding of the fluorescence signals in both in vivo and ex vivo settings.

### MPI

MPI was performed using the MOMENTUM MPI scanner (Magnetic Insight Inc., Alameda, CA, USA). Materials with different concentrations ranging from 0.2 to 1.0 mg·mL^− 1^ were tested. To examine exosome metabolism, 2D MPI was performed at 4, 8, 12, 24, 48, and 120 h after injection. Three-dimensional (3D) MPI was performed on mice 24 h post-injection on mice. The MPI/CT images were 3D reconstructed and registered using VivoQuant 4.0 software (Invicro, Boston, MA, USA). Standardised MPI signals were determined by establishing a baseline tumour signal background ratio of 100% at 4 h post-injection. This baseline was then employed for the analysis of all mice, facilitating consistent and reliable evaluation of the MPI signals across different concentrations and time points.

### External thermotherapy

For in vitro magnetic thermotherapy, SED magnetic hyperthermia procedure was executed with a variable magnetic field at 354 kHz and 30 A. A PBS solution with an iron (Fe) concentration of 1 mg/ml was utilized, and the total volume of the solution was 200 µL. Temperature changes variations were meticulously monitored for 5 min using a fibre-optic thermometer subsequent to the application of the magnetic field.

For in vitro photothermal treatment, temperature changes were measured using an infrared thermal imager (FLUKE). Different concentrations of probes were subjected to NIR laser irradiation (785 nm, 1.2 W/cm^2^, 5 min).

### Anti-tumour effect in vivo

Upon reaching a tumour volume of 80 mm^3^ on the 14th day following tumour inoculation, mice were randomly divided into three groups, each consisting of five mice. The three groups, designated as as G1, G2, and G3, were injected with PBS, SED (1 mg/mL, 50 µL), and SED (1 mg/mL, 50 µL), respectively. At 4 h post-injection, G1, G2, and G3 underwent laser light for a duration of 5 min. Additionally, G3 underwent magnetic field placement for the magnetic thermal treatment. The injection of the probe and the application of photothermal treatment occurred on the 14 and 18 days post-tumour inoculation, respectively. Magnetic field placement was performed 15 and 19 days following inoculation. Body weights were recorded on days 10, 14, 18, 22, 26,30, and 32 post-tumour inoculation. On day 32, the tumours were carefully dissected, and both tumour volume and weight data were meticulously measured using a microbalance.

### In vitro histology, prussian blue staining, and immunofluorescence staining

For Prussian blue staining, sections were sequentially immersed in xylene, absolute ethanol, and 75% alcohol, followed by thorough washing with distilled and tap water. Subsequently, the sections were stained with Prussian blue staining solution, and sealed through a dehydration process.

For fluorescence staining, paraffin sections were initially deparaffinised in water. Proteinase K working solution was then added dropwise to cover the tissue, and a membrane-breaking solution was applied to disrupt the cellular membrane. Tailored to the number of slices, precise quantities of TDT enzyme, dUTP, and buffer from the TUNEL kit were added to ensure targeted staining. Nuclei were counterstained using DAPI, and the sealed slides were subjected to observation and imaged under a fluorescence microscope, facilitating a comprehensive analysis of cellular structures.,

### Statistical analysis

Statistical analysis was performed using commercial software (GraphPad Prism 8·0; Origin 2021). The obtained results are presented as mean ± standard deviation (SD). Statistical significance was set at *P < 0.05, **P < 0.01 and ***P < 0.001, ensuring robust interpretation and differentiation of statistical significance in the reported findings.

### Electronic supplementary material

Below is the link to the electronic supplementary material.

Supplementary Material 1

## Data Availability

All data generated or analysed during the course of this study are included in this published article.

## Abbreviations

CG: Control group
EG: Experiment group
EXO: Exosome
FMI: Fluorescence molecular imaging
MPI: Magnetic particle imaging
PCa: Prostate cancer
SED: SPIONs@EXO-dye
SPION: Superparamagnetic iron oxide nanoparticles
TEM: Transmission electron microscopy
