# Supplementary material for: Integration of photomagnetic bimodal imaging to monitor an autogenous exosome loaded platform: unveiling strong targeted retention effects for guiding the photothermal and magnetothermal therapy in a mouse prostate cancer model
Source: J Nanobiotechnology. 2024 Jul 17;22:421. doi: 10.1186/s12951-024-02704-0 (PMC11253357; doi:10.1186/s12951-024-02704-0)
Supplement: Supplementary file 1 — Supplementary Material 1 [file 12951_2024_2704_MOESM1_ESM.docx]

Integration of photomagnetic bimodal imaging to monitor an autogenous exosome loaded platform: unveiling strong targeted retention effects for guiding the photothermal and magnetothermal therapy in a mouse prostate cancer model

*Songlu Liu*^a,1^, Wenting Shang^b,1^, Jian Song^c^, Qiubai Li^d^, Liang Wang^a^*

^a^Department of Radiology, Beijing Friendship Hospital, Capital Medical University Beijing 100050, China

^b^CAS Key Laboratory of Molecular Imaging, Beijing Key Laboratory of Molecular Imaging, Beijing 100190, China

^c^Department of Urology, Beijing Friendship Hospital, Capital Medical University, Beijing, China

^d^Department of Radiology, University Hospitals Cleveland Medical Center, Cleveland, Ohio, USA.

^1^Songlu Liu and Wenting Shang contributed equally to this work and share first authorship.

*Corresponding Author:

Liang Wang, MD, PhD

E-mail: 1311935212@qq.com

Telephone number: 86-10-63138140

Fax number: 86-10-63138140


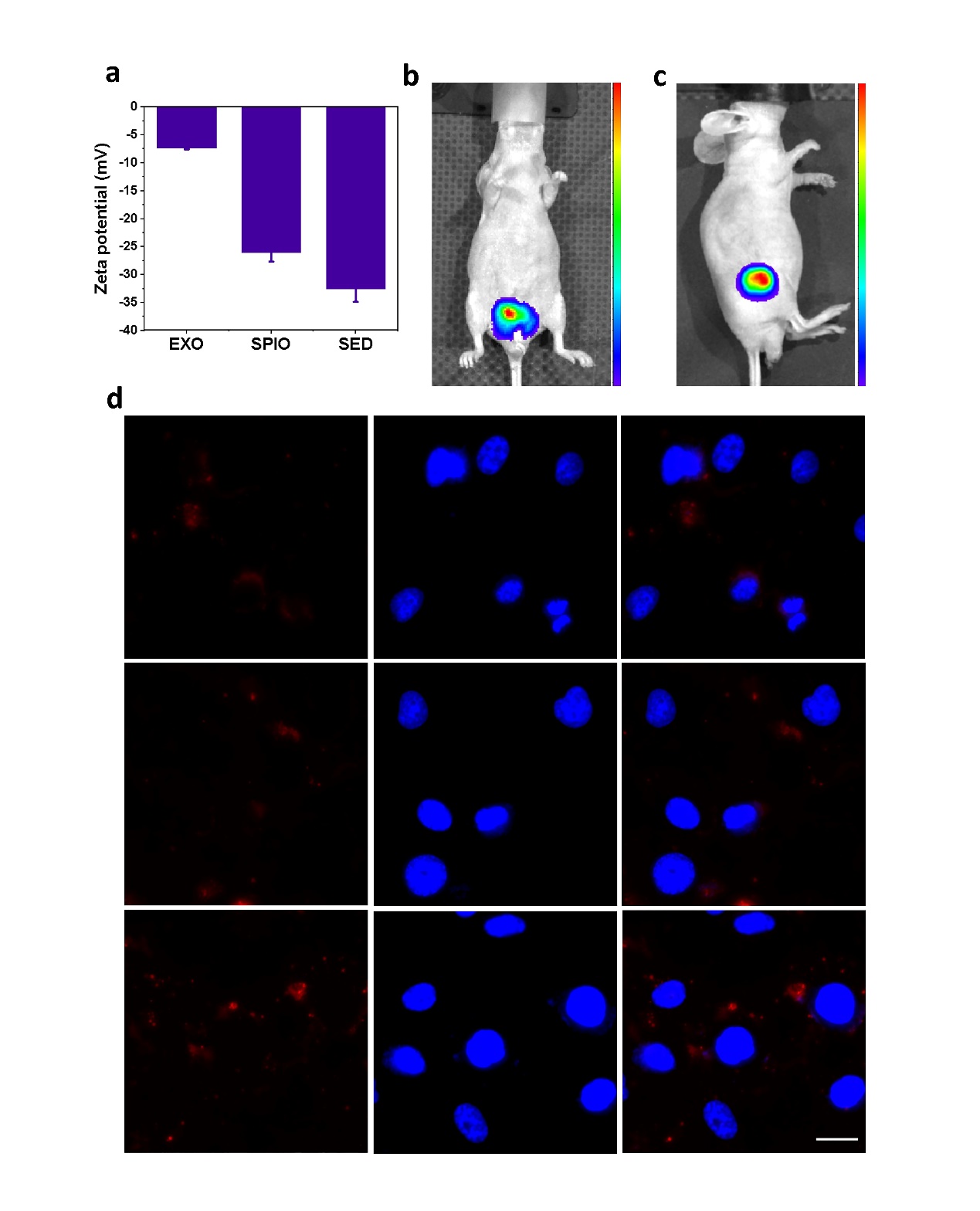


**Fig. S1.** (a) The zeta potential of the three materials. (b) BLI image of a subcutaneous tumor model of prostate cancer in mice.(c) BLI image of a orthotopic tumor model of prostate cancer in mice.(d) Cy5.5@EXO were incubated with HUVEC cell, and images were taken by Thunder imager 3D assay imaging system (scale bar: 20 μm).


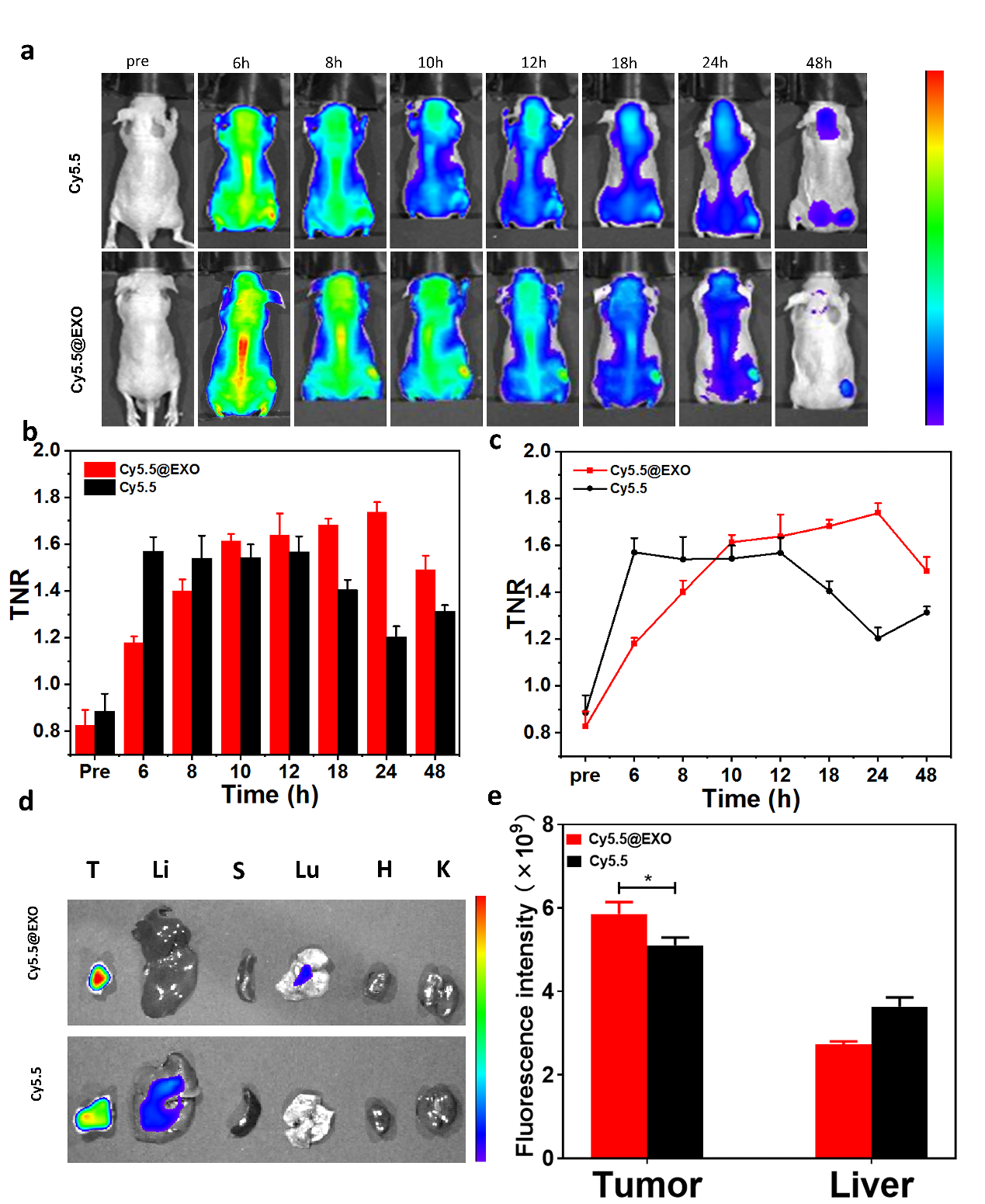


**Fig. S2.** Homologous targeting of exosome-loaded platforms. (a) Fluorescence images of PC3 subcutaneous tumor model at different time points (n=4). (b)(c) Comparison of TBR in the PC3 subcutaneous tumor model at different time points. (d) In vitro imaging of tumor tissues and major organ tissues in two groups of subcutaneous tumor models. (e) Fluorescence quantitative analysis of tumor tissue and liver tissue of the two groups of mice. Student’s *t* test. *, *P* < 0.05; **, *P* < 0.01; ***, *P* < 0.001.


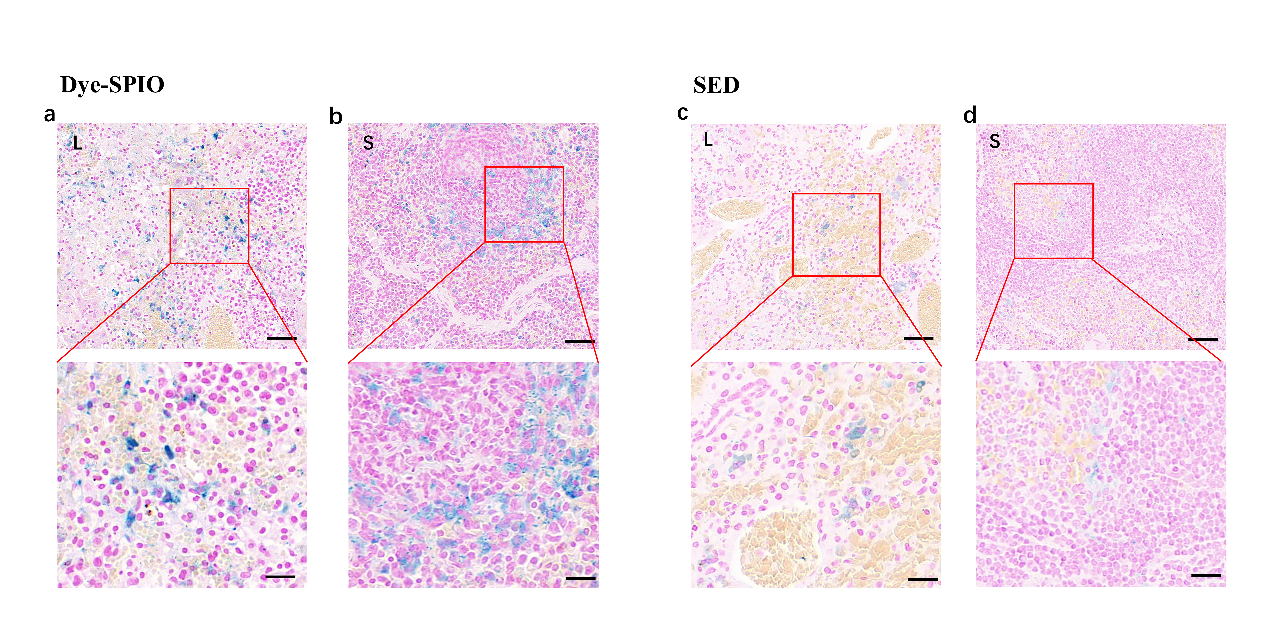


**Fig. S3.** The Prussian blue staining of (a) liver tissue in the Dye-SPIO group, (b) spleen tissue in the Dye-SPIO group, (c) liver tissue in the SED group, (d) spleen tissue in the SED group from subcutaneous tumor models. ( The scale bar of the first row:50μm; The scale bar of the second row: 20μm.)


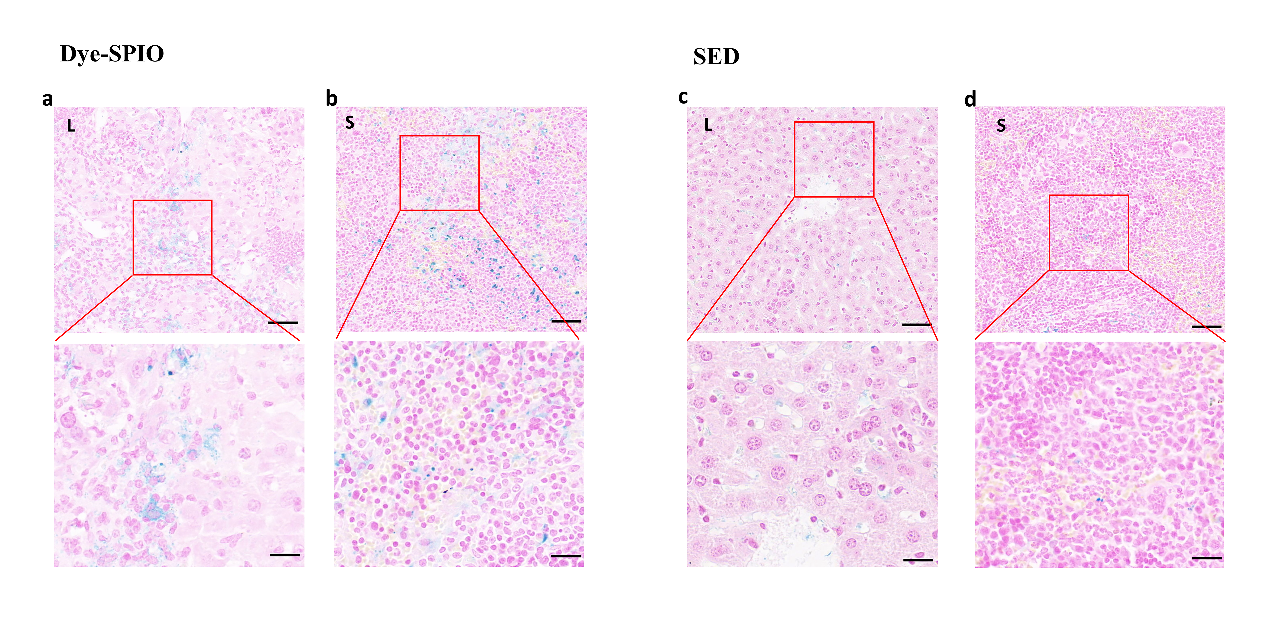


**Fig. S4.** The Prussian blue staining of (a) liver tissue in the Dye-SPIO group, (b) spleen tissue in the Dye-SPIO group, (c) liver tissue in the SED group, (d) spleen tissue in the SED group from orthotopic tumor models. ( The scale bar of the first row:50μm; The scale bar of the second row: 20μm.)


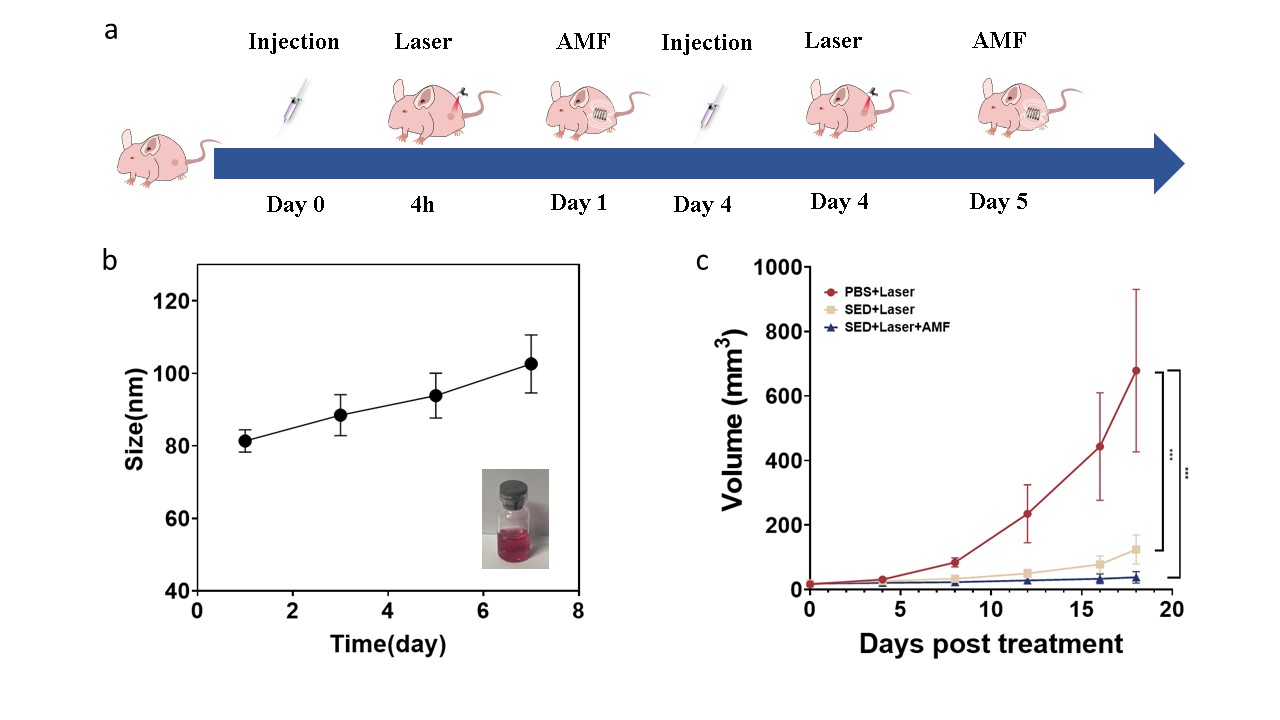


**Fig. S5.** (a) Treatment experimental plan for G3 treatment group.(b) Monitoring of hydrated particle size of SED dissolved in DMEM medium containing 10% FBS.(c) Tumor growth curves of mouse tumor models in groups G1, G2, and G3 during the monitoring period.


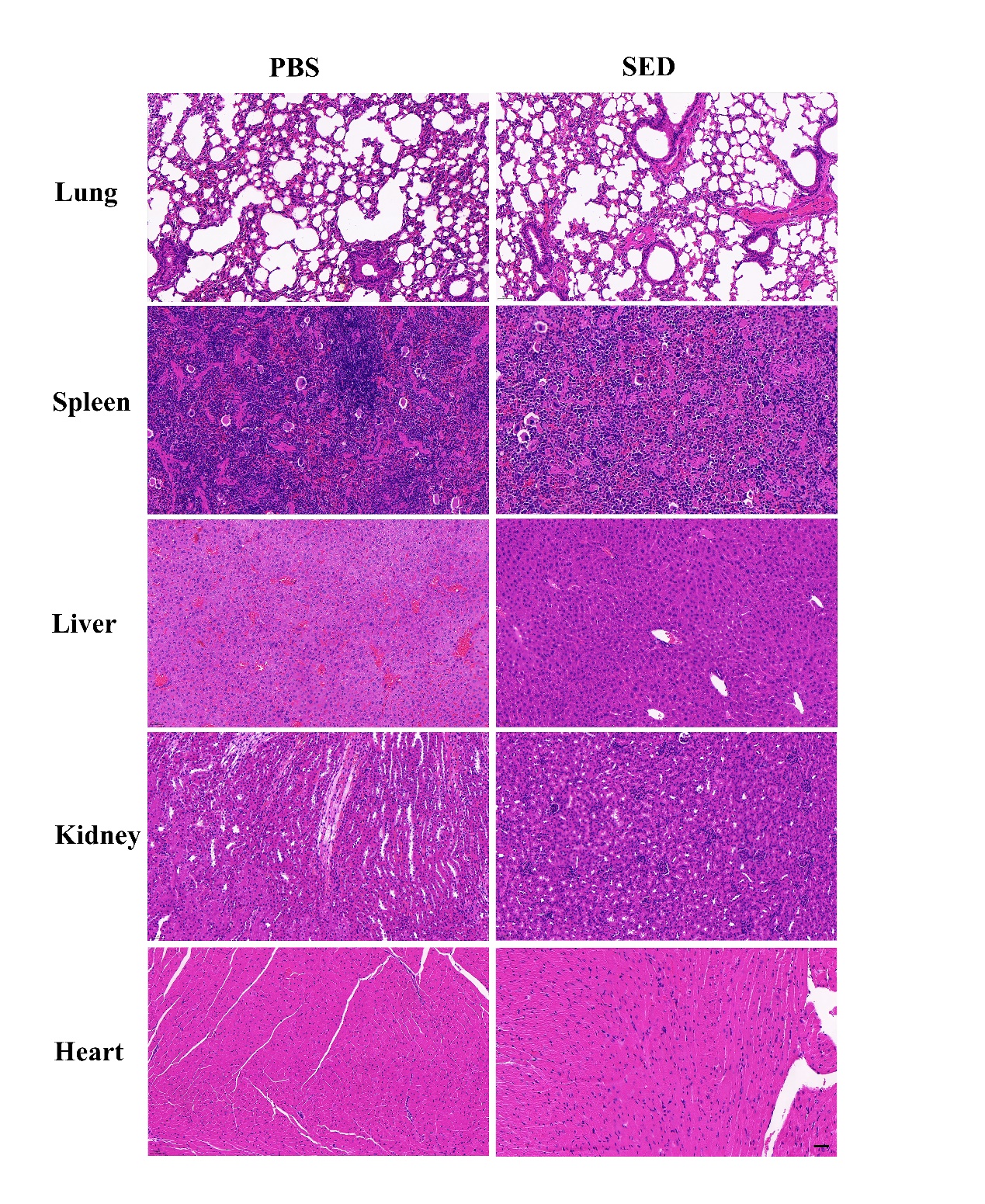


**Fig. S6.** Biocompatibility of SED nanovectors. Histology staining of tissue slices (kidney, lung, spleen, liver, and heart) obtained from different groups (PBS, SED) after treatment (Scale bar: 50μm).
